# Socioeconomic position and prediagnostic health care contacts in children with cancer in Denmark: a nationwide register study

**DOI:** 10.1186/s12885-021-08837-x

**Published:** 2021-10-14

**Authors:** Line Hjøllund Pedersen, Friederike Erdmann, Gitte Lerche Aalborg, Lisa Lyngsie Hjalgrim, Hanne Bækgaard Larsen, Kjeld Schmiegelow, Jeanette Falck Winther, Susanne Oksbjerg Dalton

**Affiliations:** 1 Department of Paediatrics and Adolescent Medicine, Rigshospitalet, Copenhagen, Denmark; 2 Survivorship and Inequality in Cancer, Danish Cancer Society Research Center, Copenhagen, Denmark; 3 Division of Childhood Cancer Epidemiology, Institute for Medical Biostatistics, Epidemiology and Informatics (IMBEI), Johannes Gutenberg University Mainz, Mainz, Germany; 4 Statistics and Data Analysis, Danish Cancer Society Research Center, Copenhagen, Denmark; 5 Institute of Clinical Medicine, University of Copenhagen, Copenhagen, Denmark; 6 Childhood Cancer, Danish Cancer Society Research Center, Copenhagen, Denmark; 7 Department of Clinical Oncology & Palliative Care, Zealand University Hospital, Naestved, Denmark

**Keywords:** Childhood, Childhood cancer, Socioeconomic position, Social inequality, Prediagnostic contacts, Denmark, Register study, Diagnosis, Stage

## Abstract

**Background:**

While underlying mechanisms and pathways of social inequalities in cancer survival have been extensively examined in adults, this is less so for children with cancer. Hypothesized mechanisms include prediagnostic utilization of and navigation through the health care system, which may differ by socioeconomic resources of the families. In this nationwide register-based study we investigated the association between measures of family socioeconomic position in relation to prediagnostic health care contacts and stage of disease at diagnosis in children with cancer in Denmark.

**Methods:**

We identified all children diagnosed with a cancer at ages 0–15 years in 1998–2016 (*N* = 3043) from the Danish Childhood Cancer Registry. We obtained comprehensive information on measures of socioeconomic position, parental health and prediagnostic contacts to both general practitioners and hospitals 24 months prior to diagnosis from various national registries. We fitted multivariable conditional logistic regression models for the association of family socioeconomic and health-related variables with firstly, frequent health care contacts and secondly, advanced stage.

**Results:**

We found higher odds ratios (OR) of frequent both overall and emergency health care contacts in the last 3 months before diagnosis in children from households with short parental education and mixed affiliation to work market, when compared to children with high family socioeconomic position. Further, children of parents with depression or of non-Western origin, respectively, had higher OR for frequent overall and emergency contacts. We found no association between socioeconomic position, parental health and stage of disease.

**Conclusion:**

Families with socioeconomic disadvantage, non-Western origin or depression more frequently utilize prediagnostic health care services, both generally and in the acute setting, indicating that some disadvantaged families may struggle to navigate the health care system when their child is sick. Reassuringly, this was not reflected in disparities in stage at diagnosis. In order to improve the diagnostic process and potentially reduce health care contacts, attention and support should be given to families with a high number of health care contacts over a short period of time.

**Supplementary Information:**

The online version contains supplementary material available at 10.1186/s12885-021-08837-x.

## Background

Although survival from childhood cancer has improved substantially over the past five decades [1], cancer remains the leading cause of disease-related deaths among 1–15 years olds in Europe [2]. Not all children benefit equally from the recent diagnostic and therapeutic improvements and large survival disparities have been observed across countries [1] as well as within countries, including European countries [3, 4] and specifically also Denmark [5], where equal access to health care services irrespective of socioeconomic position (SEP) is presumed. Lower survival has been demonstrated for children of families where parents have short education [5–7], low income [8], are single [5, 9] and have poor living conditions [10] as well as for children with siblings [5, 7, 11] and of higher birth order [11], compared to children from more advantaged families. While underlying mechanisms and pathways of social inequalities in cancer have been extensively assessed in adults, this does not hold true for childhood cancer. Despite the significant public health relevance, the underlying mechanisms of these empirically demonstrated survival inequalities in children with cancer remain poorly understood.

One hypothesized mechanism contributing to socioeconomic differences in survival refers to differences in the families social resources and coping behavior [12–14], health literacy as well as communication skills and barriers with health professionals [15–17] which may lead to differential delay in diagnosis [18]. Delay in diagnosis may be reflected in a more frequent use of health care contacts, leading to more advanced disease and potentially poorer outcome. In Denmark, general practitioners (GP) are important gatekeepers to diagnosis and treatment [19, 20]. Studies have shown a high utilization of primary care services in the prediagnostic period of children with cancer [18–20] as well as a social gradient in the utilization of GP before diagnosis [18, 19]. Beside GPs, GPs on call (emergency doctors) and emergency units may be important gatekeepers to diagnosis [21]. However, particularly the utilization of emergency services or hospital contacts in the prediagnostic period are poorly assessed for children with cancer, and such evidence may contribute to a better understanding of the causal mechanisms and pathways of social inequities in childhood cancer survival.

Stage at diagnosis is a strong predictor for cancer survival in both adult and childhood cancer [22–24]. The evidence on the prognostic impact of time to diagnosis of childhood cancer is inconsistent and heterogeneous across cancer types [25], but is of significant concern as early diagnosis may still prevent progression and secure less extensive treatment. More advanced disease at diagnosis requiring a greater burden of cancer therapy in children with Wilms tumor was observed in the UK, where the health care system is also characterized by GPs acting as gatekeepers to specialized health care [26]. In a recent review, Mogensen et al. [3] suggested a potential association between SEP and advanced stage of disease at diagnosis [23, 27, 28] but emphasized that findings were contradictory [22, 29, 30]. To our knowledge, no previous study has addressed the association between SEP, prediagnostic health care contacts and stage at diagnosis in children with cancer. In this nationwide study, we took advantage of the high-quality registry data of Denmark to examine the association between SEP and the number of health care contacts during the 24 months prior to a childhood cancer diagnosis with a particular focus on the last 3 months before diagnosis. Moreover, in a subpopulation, we assessed the association between SEP and stage of disease at diagnosis and examined whether number of health care contacts may mediate such an association.

## Methods

Denmark has a population-based register infrastructure with long-standing administrative registries with high-quality health and socioeconomic data and a unique personal identification number used in all registries [31] that enables individual linkage of information across registries. Linkage of information across registries provided the basis for our nationwide register study.

### Study design and population

We identified all incident cases of cancer diagnosed in children aged 0–15 years in 1998–2016 in Denmark from the Danish Childhood Cancer Registry (DCCR) [32]. The DCCR is a nationwide clinical quality database set up to monitor the quality of childhood cancer care in Denmark. For each child with cancer, we obtained information on date of diagnosis, diagnosis code and stage of disease from the DCCR. Diagnoses were coded according to the International Classification of Childhood Cancer third version (ICCC-3) into 12 major diagnostic groups. We defined the following five cancer groups based on the ICCC-3 major diagnostic groups [33]: Leukemias, myeloproliferative diseases, and myelodysplastic diseases (Leukemia), Lymphomas and reticuloendothelial neoplasms (Lymphoma), CNS and miscellaneous intracranial and intraspinal neoplasms (CNS tumors), Malignant bone tumor and soft tissue and other extraosseous sarcomas (Bone tumors and soft tissue sarcomas), and other non-CNS solid tumors. With this grouping, we aggregated cancer types with similar characteristics into groups of larger sample sizes to increase statistical power, while keeping distinct diagnostic groups separate for meaningful analysis.

### Socioeconomic position of the household

By using the personal identification number from the Danish Civil Registration System [31], we were able to identify the respective household for each child and the parents they were living with at time of diagnosis. In the following denoted as household parents regardless of biological relationship.

Information from the social registries administered at Statistics Denmark were used to define the SEP of the household [34–36]. SEP is a multidimensional construct and determined by various indicators measuring different aspects of economic and social resources, assets and conditions [37]. Indicators of SEP included highest parental education in the household categorized into short (< 10 years, primary and lower secondary education), medium (10–12 years, upper secondary and vocational) and long education (> 12 years, higher education); household cohabitation status categorized into parents living together or living alone; highest parental annual disposable income categorized into quintiles (based on the sex, birth cohort and calendar year-specific income distribution of the entire Danish population); household affiliation to work market categorized into at work, unemployed/not in work force and mixed (household parents belong to different categories); country of origin for household parents categorized into Denmark, Western country, non-Western country and mixed (household parents belong to different categories); number of children in household categorized into one, two or three or more. Information on education, income, affiliation to the work market were obtained the year prior to diagnosis and for cohabitation status and number of children for the year of diagnosis.

### Health of the household parents

We obtained information on chronic somatic diseases included in the Charlson Comorbidity Index [38] and diagnosis of a major psychiatric disorder (not including depression) (ICD-8: 290 to 295.99, 296.19, 296.39, 303.00–304.99 and ICD-10: F00-F31) in the household parents for the 5 years prior to the child’s cancer diagnosis from the Danish National Patient Register (NPR) [39] and the Danish Psychiatric Central Research Register [40] and created a variable for somatic or major psychiatric disease in household parents, categorized as yes (one or more disease) or no (none). We further included information from the Danish National Prescription Registry [41] to create a variable describing depression in the household parents during 2 years prior to the cancer diagnosis (yes/no) based on any redeemed prescription for antidepressants (ATC-group N06A) or any diagnosis of unipolar depression (ICD-8: 296.09, 296.29 or ICD-10: F32-F33.9).

### Health care contacts

The Danish National Health Service Register holds information on all contacts and procedures/services in the primary health care sector in Denmark since 1990 [42], while the NPR has registered all hospital admissions since 1978 and outpatient and emergency contacts since 1994 [39]. Information on all health care contacts including date and contact type were collected for 24 months prior to the child’s diagnosis. From the Danish National Health Service Register, we collected all GP contacts including face-to-face, e-mail consultations and home visits. Contacts in relation to the Danish mandatory pediatric examination program (yearly screens for development and thriving) and the national vaccination program were not considered. We also obtained information on contacts to private practicing doctors other than GPs. Finally, we obtained information on consultations with GPs on call. From the NPR, we collected information on all contacts to elective outpatient departments, in-patient hospitalizations and to emergency departments. We created the variable ‘all contacts’ which included all primary and secondary health care contacts for the time windows 0–24 months, 19–24 months, 13–18 months, 7–12 months, 4–6 months and 0–3 months prior to diagnosis, and respectively for ‘emergency contacts’ that included all GP on call contacts, emergency room contacts and acute hospitalizations for the equivalent time windows. Based on the distribution of health care contacts throughout the full period of 24 months prior to diagnosis, we defined the period of primary interest as the period immediately up to diagnosis. Due to a similar pattern across all diagnostic groups, this was defined as the last 3 months prior to diagnosis. For this period, we classified contacts into frequent and less frequent based on the median number of contacts for all contacts and emergency contacts.

### Stage

We defined stage individually by cancer type based on clinical considerations, as different cancer types are biologically very heterogeneous. The availability of appropriate clinical information was limited to certain cancer types and included B-cell acute lymphoblastic leukemia (ALL), T- cell ALL, Hodgkin lymphoma, non-Hodgkin and Burkitt lymphoma, Ewing’s sarcoma, osteosarcoma and rhabdomyosarcoma. Based on data availability, we used either the Toronto Pediatric Cancer Staging criteria [43] or similar measure as available from the DCCR. For ALL, we categorized white blood cell count at diagnosis (WBC) <  100 × 10^9^/L as low stage and WBC ≥ 100 × 10^9^/L as advanced stage. For lymphoma, we categorized stage 1–2 as low stage and stage 3–4 as advanced stage. For the groups of bone tumors and soft tissue sarcomas, we categorized stage as no distant metastases/unknown as low stage and distant metastases as advanced stage.

### Statistical analyses

Descriptive analyses were performed to describe the number of children diagnosed with cancer at ages 0–15 years in total and by highest parental education in the household. To investigate the number of contacts in the 24 months prior to diagnosis, we plotted the number of contacts by time before diagnosis for each of the defined diagnostic groups.

Logistic regression was used to estimate multivariate-adjusted odds ratios (OR) and 95% confidence intervals (CI) for the association between selected socioeconomic variables and the odds of frequent contacts to the health care system in the last 3 months before diagnosis for all contacts and emergency contacts respectively. Two models were used. In the first model we adjusted for diagnostic group, sex, period of diagnosis and age at diagnosis (as a continuous variable), and in the second model, we additionally adjusted for highest parental education in household and household cohabitation status. We analyzed each SEP variable separately, however, adjusted for education and cohabitation status as we understand them as potential confounders on the association between other SEP indicators and health care use. To assess effect modification, we conducted stratified analyses by diagnostic groups, sex and age at diagnosis (0, 1–4, 5–10, 11–15), including the variable in question as an interaction term.

Finally, we considered the association between highest parental education in the household, household cohabitation status and depression in household parents and disease stage in a subpopulation. Logistic regression was used to estimate multivariate-adjusted ORs and 95% CI for the association between the selected socioeconomic variables and the odds of advanced stage. Again, two models were used. In the first model we adjusted for sex, period of diagnosis and age at diagnosis (as a continuous variable), highest parental education in the household and household cohabitation, and in the second model we additionally adjusted for total number of contacts in the 24 months before diagnosis (as a continuous variable). In both models diagnostic subgroup was included as an interaction term.

Statistical significance was considered at a 5% level, and all statistical analyses were conducted using the statistical software R, version 3.6.3.

## Results

### Study population

From our initial sample of 3115 children, we excluded 26 children who were living in own households or with no adults and 18 with missing information on affiliation to work market and/or income. Furthermore, we excluded 28 children diagnosed on their date of birth, resulting in a final study population of 3043 children diagnosed with cancer. The most common diagnoses included leukemia (30%) and CNS tumors (27%). Almost half of the children were diagnosed before the age of 5 years (45%) (Table 1). About 45% of the children were living in families with long and 12% with short parental education. Compared to families with medium or long parental education, families with short parental education were more often young (mothers ≤30 years, 38%), had low income (14% Q1 and 28% Q2), were unemployed (42%), were single parents (46%), originated from a non-Western country (26%), and had a depression (19%) compared to families with medium or long parental education.

**Table 1:** Characteristics of the study population by highest parental education in the household

Characteristics	Highest parental education in the household^**a**^			Total
	Short	Medium	Long	
	N (%)	N (%)	N (%)	N (%)
**No. of patients (1998–2016)**	354 (100)	1316 (100)	1373 (100)	3043 (100)
**Age at diagnosis (years)**				
0	41 (12)	132 (10)	131 (10)	304 (10)
1-4	123 (35)	417 (32)	534 (39)	1074 (35)
5-10	113 (32)	390 (30)	394 (29)	897 (29)
11-15	77 (22)	377 (29)	314 (23)	768 (25)
**Sex**				
Female	151 (43)	599 (46)	672 (49)	1422 (47)
Male	203 (57)	717 (54)	701 (51)	1621 (53)
**Period of diagnosis**				
1998–2001	81 (23)	350 (27)	245 (18)	676 (22)
2002–2006	98 (28)	368 (28)	332 (24)	798 (26)
2007–2011	90 (25)	326 (25)	387 (28)	803 (26)
2012–2016	85 (24)	272 (21)	409 (30)	766 (25)
**Diagnostic group**				
Leukemia	103 (29)	382 (29)	420 (31)	905 (30)
Lymphoma	43 (12)	135 (10)	136 (10)	314 (10)
CNS tumors	88 (25)	362 (28)	355 (26)	805 (26)
Bone tumors and soft tissue sarcomas	39 (11)	139 (11)	143 (10)	321 (11)
Other non-CNS solid tumors	81 (23)	298 (23)	319 (23)	698 (23)
**Age of mother at time of diagnosis**				
≤ 25	59 (17)	70 (5)	12 (1)	141 (5)
26–30	75 (21)	204 (16)	157 (11)	436 (14)
31–35	93 (26)	340 (26)	387 (28)	820 (27)
36–40	70 (20)	356 (27)	428 (31)	854 (28)
≥ 41	46 (13)	325 (25)	372 (27)	743 (24)
Household without mother	11 (3)	21 (2)	17 (1)	49 (2)
**Age of father at time of diagnosis**				
≤ 30	51 (14)	172 (13)	90 (7)	313 (10)
31–35	45 (13)	250 (19)	300 (22)	595 (20)
36–40	48 (14)	282 (21)	378 (28)	708 (23)
≥ 41	60 (17)	437 (33)	516 (38)	1013 (33)
Household without father	150 (42)	175 (13)	89 (6)	414 (14)
**Household cohabitation status**				
Living as a couple (with unknown relation)	193 (55)	1120 (85)	1267 (92)	2580 (85)
Living alone (single parent)	161 (45)	196 (15)	106 (8)	463 (15)
**Highest parental income in household**^**b**^				
Fifth quintile	70 (20)	275 (21)	579 (42)	924 (30)
Fourth quintile	56 (16)	365 (28)	440 (32)	861 (28)
Third quintile	79 (22)	357 (27)	221 (16)	657 (22)
Second quintile	98 (28)	231 (18)	103 (8)	432 (14)
First quintile	51 (14)	88 (7)	30 (2)	169 (6)
**Household affiliation to work market**^**c**^				
At work	133 (38)	947 (72)	1162 (85)	2242 (74)
Mixed	74 (21)	266 (20)	164 (12)	504 (17)
Unemployed/not in work force	147 (42)	103 (8)	47 (3)	297 (10)
**Country of origin for household parent(s)**^**d**^				
Denmark	243 (68)	1131 (86)	1213 (88)	2586 (85)
Western countries	9 (3)	14 (1)	20 (1)	43 (1)
Non-Western countries	93 (26)	133 (10)	69 (5)	295 (10)
Mixed	10 (3)	38 (3)	71 (5)	119 (4)
**Number of children in household**				
1	94 (27)	326 (25)	291 (21)	711 (23)
2	145 (41)	625 (47)	708 (52)	1478 (49)
≥ 3	115 (32)	365 (28)	374 (27)	854 (28)
**Depression in household parent(s)**				
No	288 (81)	1132 (86)	1248 (91)	2668 (88)
Yes	66 (19)	184 (14)	125 (9)	375 (12)
**Somatic or major psychiatric disease in household parent(s)**				
None	310 (88)	1182 (90)	1247 (91)	2739 (90)
One or more	44 (12)	134 (10)	126 (9)	304 (10)

^a^Highest parental education in the household categorized into short (< 10 years, primary and lower secondary education), medium (10–12 years, upper secondary and vocational education) and higher education (≥3 years, higher education)

^b^Highest parental income in household categorized into quintiles with first quintile as the lowest (< 20%). This is based on the highest annual disposable parental income in household based on the income quintile of the entire Danish population by birth cohort, calendar year and sex

^c^Household affiliation to work market categorized into at work, unemployed/not in work force and mixed (one household parent at work and one unemployed/not in work force)

^d^Country of origin of household parents categorized into Denmark, Western country, Non-western country and mixed (household parents belong to different country categories)

### Contacts with the health care system

Among all childhood cancer patients, children diagnosed with leukemia had the highest number of all contacts (all primary and secondary health care contacts) and emergency contacts (all GP on call contacts, emergency room contacts and acute hospitalizations) to the health care system throughout the 24 months as well as in the 3 months before diagnosis (Supplementary Table 1).

**Fig. 1:**
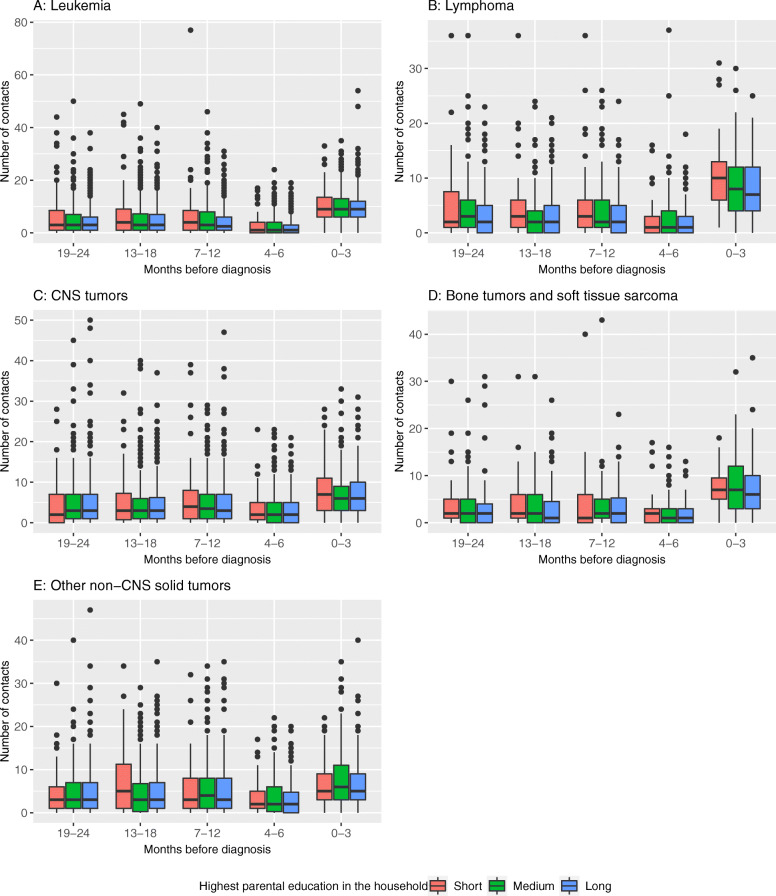
Distribution of pre-diagnostic contacts to the health care system in children diagnosed with cancer 0–24 months before diagnosis by highest parental education in the household, diagnostic group and time since diagnosis. The length of the box represents the interquartile range, the horizontal black line in the box interior represents the median, and the dots represent outliers

Number of contacts were particular high during the last 3 months prior to diagnosis across all diagnostic groups. For this period, 47% of the 3043 children showed frequent contacts (≥8 contacts) to the health care system and 43% had frequent emergency contacts (≥2 emergency contacts). Figure 1 shows the median number of contacts by time before diagnosis and by parental education for the diagnostic groups.

While there is no clear pattern by education over time from 24 to 4 months before diagnosis across diagnostic groups, there is an indication that children living in families with short parental education and diagnosed with leukemia, lymphoma or CNS tumors had more contacts in the last 3 months prior to diagnosis than children from families with medium or long parental education. Similar plots for household cohabitation status indicated no differences in number of contacts over the 24 months prior to diagnosis (data not shown).

### Frequent contacts with the health care system

Analyzing specifically the last 3 months prior to diagnosis, children from families with short parental education had higher ORs of frequent contacts to the health care system across diagnostic groups compared to families with long education (Table 2), although only statistically significant for CNS tumors (OR: 1.81; 95% CI: 1.12–2.93). Similarly increased ORs of frequent contacts across diagnostic groups were observed in children from families with mixed affiliation to the work market compared to working parents, reaching statistical significance for leukemia (OR: 1.80; 95% CI: 1.22–2.66) as well as for other non-CNS solid tumors (OR: 1.97; 95% CI: 1.34–2.91). Lower ORs were seen for children of single parents for all diagnostic groups with the exception of bone tumors and soft tissue sarcomas; however, none of them reached statistical significance. Depression in household parents was (with the exception of children with lymphoma) associated with elevated ORs for frequent contacts, reaching statistical significance in children with CNS tumors (OR: 1.56; 95% CI: 1.04–2.34). We found no clear pattern of associations with income, number of children in household and somatic and major psychiatric disease in parents.

**Table 2:** Odds ratio (95% CI) of frequent contacts to the health care system (all contacts)

	Leukemia	Lymphoma	CNS tumors	Bone tumors and soft tissue sarcomas	Other non-CNS solid tumors	All cancers
	(***N*** = 905)	(***N*** = 314)	(***N*** = 805)	(***N*** = 321)	(***N*** = 698)	(***N =*** 3043)
	OR (95% CI)	OR (95% CI)	OR (95% CI)	OR (95% CI)	OR (95% CI)	OR (95% CI)
**Highest parental education in household**						
Long	1 (Ref.)	1 (Ref.)	1 (Ref.)	1 (Ref.)	1 (Ref.)	1 (Ref.)
Medium	1.07 (0.81–1.43)	1.60 (0.99–2.59)	1.10 (0.81–1.49)	1.22 (0.76–1.96)	1.29 (0.93–1.78)	1.19 (1.01–1.39)
Short	1.16 (0.74–1.83)	1.96 (0.96–3.99)	1.81 (1.12–2.93)	1.31 (0.64–2.71)	1.23 (0.74–2.05)	1.42 (1.10–1.83)
**Household cohabitation status**						
Parents as couple	1 (Ref.)	1 (Ref.)	1 (Ref.)	1 (Ref.)	1 (Ref.)	1 (Ref.)
Single parent	0.86 (0.58–1.27)	0.85 (0.47–1.55)	0.68 (0.45–1.03)	1.09 (0.59–2.01)	0.91 (0.58–1.43)	0.84 (0.68–1.04)
**Highest parental income in household**						
Fifth quintile	1 (Ref.)	1 (Ref.)	1 (Ref.)	1 (Ref.)	1 (Ref.)	1 (Ref.)
Fourth quintile	1.74 (1.24–2.46)	1.73 (0.95–3.15)	1.03 (0.71–1.50)	0.86 (0.49–1.53)	0.98 (0.65–1.48)	1.24 (1.02–1.50)
Third quintile	1.40 (0.95–2.07)	1.91 (0.99–3.70)	0.71 (0.48–1.07)	1.85 (0.96–3.57)	1.19 (0.78–1.81)	1.20 (0.97–1.49)
Second quintile	1.94 (1.25–3.01)	1.75 (0.84–3.62)	0.97 (0.62–1.54)	0.77 (0.38–1.58)	1.19 (0.71–1.98)	1.30 (1.02–1.66)
First quintile	2.71 (1.34–5.48)	1.10 (0.41–2.90)	0.60 (0.30–1.22)	0.95 (0.30–2.96)	1.01 (0.51–2.00)	1.16 (0.82–1.64)
**Household affiliation to work market**						
At work	1 (Ref.)	1 (Ref.)	1 (Ref.)	1 (Ref.)	1 (Ref.)	1 (Ref.)
Mixed	1.80 (1.22–2.66)	1.68 (0.86–3.25)	1.19 (0.79–1.78)	1.24 (0.66–2.34)	1.97 (1.34–2.91)	1.59 (1.29–1.95)
Unemployed/not in work force	0.71 (0.44–1.16)	0.88 (0.43–1.80)	1.04 (0.63–1.73)	1.06 (0.51–2.20)	1.27 (0.74–2.20)	0.96 (0.72–1.28)
**Country of origin for household parent(s)**						
Denmark	1 (Ref.)	1 (Ref.)	1 (Ref.)	1 (Ref.)	1 (Ref.)	1 (Ref.)
Western countries	0.71 (0.27–1.88)	^a^	2.09 (0.46–9.56)	2.05 (0.33–12.62)	0.47 (0.13–1.72)	0.91 (0.49–1.70)
Non-Western countries	1.12 (0.68–1.83)	^a^	1.01 (0.61–1.67)	1.76 (0.85–3.65)	0.97 (0.58–1.61)	1.02 (0.79–1.32)
Mixed	0.63 (0.34–1.18)	^a^	0.68 (0.33–1.41)	2.05 (0.54–7.86)	0.40 (0.15–1.09)	0.71 (0.48–1.04)
**Number of children in household**						
1	1 (Ref.)	1 (Ref.)	1 (Ref.)	1 (Ref.)	1 (Ref.)	1 (Ref.)
2	1.12 (0.81–1.56)	0.89 (0.46–1.73)	0.82 (0.57–1.18)	0.76 (0.42–1.40)	1.21 (0.83–1.75)	1.01 (0.84–1.22)
≥ 3	1.18 (0.81–1.72)	0.58 (0.28–1.20)	0.81 (0.54–1.22)	0.65 (0.34–1.22)	1.16 (0.76–1.76)	0.94 (0.76–1.16)
**Depression in household parents**						
No	1 (Ref.)	1 (Ref.)	1 (Ref.)	1 (Ref.)	1 (Ref.)	1 (Ref.)
Yes	1.34 (0.87–2.06)	0.92 (0.45–1.85)	1.56 (1.04–2.34)	1.56 (0.80–3.04)	1.12 (0.70–1.81)	1.32 (1.05–1.65)
**Somatic or major psychiatric disease in household parents**						
No	1 (Ref.)	1 (Ref.)	1 (Ref.)	1 (Ref.)	1 (Ref.)	1 (Ref.)
Yes	1.38 (0.87–2.19)	1.80 (0.92–3.53)	0.89 (0.52–1.52)	0.60 (0.28–1.29)	1.11 (0.67–1.83)	1.13 (0.88–1.44)

Frequent contacts are defined as 8 or more contacts to doctors in primary care sector, emergency room contacts, outpatient and inpatient hospital contacts during the last 3 months before child’s cancer diagnosis; *OR* Odds Ratio, *CI* confidence interval. For each socioeconomic variable, the models are adjusted for sex, diagnostic group (as an interaction term in the model, where we estimate the OR for each diagnostic group), diagnosis period and age at diagnosis (continuous), highest parental education in the household and household cohabitation status. ^a^ Due to few observations, logistic regression analysis did not reach convergence.

For emergency contacts (Table 3), we similarly observed higher OR of frequent emergency contacts among children from families with short parental education compared to households with long education across most diagnostic groups, however, only statistically significant for lymphoma (OR: 2.59; 95% CI: 1.27–5.29). ORs for most diagnostic groups were also higher among children from families with mixed affiliation to work market and with parents of non-Western origin, although confidence intervals were in general wide. A distinct association between depression in household parents and frequent emergency contacts was found among children with CNS tumors (OR: 2.13; 95% CI: 1.41–3.23), while ORs for children from households with parental somatic or major psychiatric disease were increased for leukemia and CNS tumors, albeit not statistically significant. Conversely, somatic or major psychiatric disease was associated with an OR of 0.36 (95% CI: 0.14; 0.98) among children with bone tumors and soft tissue sarcomas. No obvious pattern in ORs was noted for cohabitation status, income and number of children in household.

**Table 3:** Odds ratio (95% CI) of frequent emergency contacts to the health care system

	Leukemia	Lymphoma	CNS tumors	Bone tumors and soft tissue sarcomas	Other non-CNS solid tumors	All cancers
	(***N =*** 905)	(***N =*** 314)	(***N =*** 805)	(***N =*** 321)	(***N =*** 698)	(***N =*** 3043)
	OR (95% CI)	OR (95% CI)	OR (95% CI)	OR (95% CI)	OR (95% CI)	OR (95% CI)
**Highest parental education in household**						
Long	1 (Ref.)	1 (Ref.)	1 (Ref.)	1 (Ref.)	1 (Ref.)	1 (Ref.)
Medium	1.02 (0.76–1.37)	0.98 (0.59–1.61)	1.22 (0.89–1.68)	0.81 (0.48–1.36)	0.98 (0.70–1.38)	1.03 (0.87–1.21)
Short	1.03 (0.64–1.64)	2.59 (1.27–5.29)	1.62 (0.99–2.66)	1.31 (0.61–2.79)	1.19 (0.72–1.99)	1.39 (1.07–1.80)
**Household cohabitation status**						
Parents as couple	1 (Ref.)	1 (Ref.)	1 (Ref.)	1 (Ref.)	1 (Ref.)	1 (Ref.)
Single parent	0.88 (0.59–1.32)	0.95 (0.51–1.74)	1.02 (0.68–1.53)	1.89 (1.00–3.57)	0.94 (0.59–1.49)	1.02 (0.81–1.27)
**Highest parental income in household**						
Fifth quintile	1 (Ref.)	1 (Ref.)	1 (Ref.)	1 (Ref.)	1 (Ref.)	1 (Ref.)
Fourth quintile	1.14 (0.80–1.63)	1.12 (0.60–2.10)	1.21 (0.81–1.79)	1.07 (0.58–1.97)	0.88 (0.58–1.34)	1.08 (0.88–1.31)
Third quintile	0.90 (0.60–1.34)	1.55 (0.79–3.04)	1.10 (0.72–1.66)	0.81 (0.39–1.68)	1.00 (0.66–1.54)	1.03 (0.82–1.28)
Second quintile	0.79 (0.51–1.23)	1.28 (0.61–2.70)	1.13 (0.70–1.82)	0.87 (0.40–1.90)	0.74 (0.43–1.26)	0.91 (0.70–1.17)
First quintile	1.22 (0.61–2.44)	1.58 (0.59–4.23)	0.79 (0.38–1.65)	1.19 (0.36–3.92)	0.97 (0.49–1.94)	1.06 (0.74–1.51)
**Household affiliation to work market**						
At work	1 (Ref.)	1 (Ref.)	1 (Ref.)	1 (Ref.)	1 (Ref.)	1 (Ref.)
Mixed	1.31 (0.89–1.93)	0.88 (0.45–1.72)	1.08 (0.71–1.64)	1.60 (0.82–3.11)	1.48 (1.00–2.20)	1.26 (1.02–1.56)
Unemployed/not in work force	0.86 (0.52–1.43)	1.32 (0.64–2.72)	0.91 (0.53–1.54)	1.80 (0.85–3.81)	1.09 (0.62–1.91)	1.06 (0.79–1.42)
**Country of origin for household parent(s)**						
Denmark	1 (Ref.)	1 (Ref.)	1 (Ref.)	1 (Ref.)	1 (Ref.)	1 (Ref.)
Western countries	1.53 (0.49–4.79)	^a^	11.50 (1.35–97.64)	1.62 (0.26–10.03)	0.86 (0.26–2.85)	1.82 (0.94–3.50)
Non-Western countries	1.37 (0.80–2.32)	^a^	1.05 (0.63–1.75)	2.07 (0.99–4.33)	2.10 (1.27–3.49)	1.49 (1.15–1.94)
Mixed	0.66 (0.35–1.26)	^a^	0.56 (0.25–1.25)	3.65 (0.95–14.04)	1.71 (0.75–3.88)	0.93 (0.62–1.38)
**Number of children in household**						
1	1 (Ref.)	1 (Ref.)	1 (Ref.)	1 (Ref.)	1 (Ref.)	1 (Ref.)
2	0.83 (0.58–1.17)	0.76 (0.39–1.47)	1.15 (0.79–1.68)	0.63 (0.33–1.19)	0.95 (0.65–1.40)	0.93 (0.76–1.13)
≥ 3	0.93 (0.62–1.38)	0.63 (0.30–1.33)	0.59 (0.38–0.92)	0.68 (0.35–1.32)	1.74 (1.14–2.65)	0.92 (0.74–1.14)
**Depression in household parents**						
No	1 (Ref.)	1 (Ref.)	1 (Ref.)	1 (Ref.)	1 (Ref.)	1 (Ref.)
Yes	1.02 (0.66–1.57)	1.08 (0.53–2.22)	2.13 (1.41–3.23)	1.29 (0.63–2.61)	0.97 (0.59–1.59)	1.30 (1.03–1.64)
**Somatic or major psychiatric disease in household parents**						
No	1 (Ref.)	1 (Ref.)	1 (Ref.)	1 (Ref.)	1 (Ref.)	1 (Ref.)
Yes	1.28 (0.80–2.06)	0.97 (0.50–1.90)	1.43 (0.84–2.42)	0.36 (0.14–0.98)	1.00 (0.59–1.69)	1.07 (0.83–1.37)

Frequent contacts are defined as 2 or more contacts to GPs on call in primary care sector and emergency room contacts during the last 3 months before child’s cancer diagnosis; *OR* Odds Ratio, *CI* confidence interval. For each socioeconomic variable, the models are adjusted for sex, diagnostic group (as an interaction term in the model, where we estimate the OR for each diagnostic group), diagnosis period and age at diagnosis (continuous), highest parental education in the household and household cohabitation status. ^a^ Due to few observations, logistic regression analysis did not reach convergence.

Stratified analysis of the total sample by sex and age group revealed that increased OR of frequent contacts for children in families with short parental education compared to long education was more pronounced among boys (OR: 1.68; 95% CI: 1.20–2.34) than girls (OR: 1.12; 95% CI: 0.77–1.63), while the association of depression in household parent with OR of frequent emergency contacts seemed stronger among girls (OR: 1.60; 95% CI: 1.14–2.25) than among boys (OR: 1.09; 95% CI: 0.80–1.49) (data not shown). In general, the ORs were higher among children from families with mixed affiliation to work market compared to working parents across age groups, however, reaching statistical significance only in infants (OR: 2.32, 95% CI: 1.31–4.10) and the youngest children (OR: 1.77, 95% CI: 1.29–2.44) (data not shown).

### Stage of disease

The sample for analyses of stage in selected cancer types included a total of 1074 children (Table 4). There was no clear pattern of an association between highest education in household parents, household cohabitation status or depression in household parent with advanced stage for the selected cancer types except for B-cell ALL, where the OR of advanced stage was increased among children of single parent households (OR: 2.39; 95% CI: 1.14–4.99) compared to cohabiting parents (Table 4). Adjusting for number of health care contacts did not change the estimates considerably (Table 4), nor did excluding age from the models.

**Table 4:** Odds ratio (95% CI) of advanced stage of disease at diagnosis in 1074 children

	Advanced stage	Low stage	Model 1^**a**^	Model 2^**b**^
**B-cell acute lymphoblastic leukemia**^**c**^ **(*N*** **= 601)**				
**Highest parental education in household**				
Long	20	270	1 (Ref.)	1 (Ref.)
Medium	18	233	1.07 (0.55–2.08)	1.09 (0.56–2.13)
Short	5	55	1.01 (0.35–2.92)	1.07 (0.37–3.08)
**Household cohabitation status**				
Parents as couple	31	480	1 (Ref.)	1 (Ref.)
Single parent	12	78	2.39 (1.14–4.99)	2.36 (1.13–4.95)
**Depression in household parents**				
No	NA	503	1 (Ref.)	1 (Ref.)
Yes	NA	55	0.64 (0.19–2.15)	0.68 (0.20–2.30)
**T-cell acute lymphoblastic leukemia**^**c**^ **(*N*** **= 98)**				
**Highest parental education in household**				
Long	19	21	1 (Ref.)	1 (Ref.)
Medium	20	27	0.83 (0.35–1.96)	0.84 (0.35–1.98)
Short	5	6	0.86 (0.22–3.35)	0.92 (0.24–3.60)
**Household cohabitation status**				
Parents as couple	38	46	1 (Ref.)	1 (Ref.)
Single parent	6	8	0.99 (0.31–3.19)	0.98 (0.30–3.18)
**Depression in household parents**				
No	37	44		1 (Ref.)
Yes	7	10	0.89 (0.30–2.62)	0.85 (0.29–2.51)
**Hodgkin lymphoma**^**d**^ **(*N*** **= 89)**				
**Highest parental education in household**				
Long	NA	30	1 (Ref.)	1 (Ref.)
Medium	NA	24	1.42 (0.57–3.53)	1.60 (0.63–4.03)
Short	NA	4	0.45 (0.04–4.46)	0.52 (0.05–5.21)
**Household cohabitation status**				
Parents as couple	26	49	1 (Ref.)	1 (Ref.)
Single parent	5	9	1.17 (0.35–3.93)	1.13 (0.34–3.78)
**Depression in household parents**				
No	26	53	1 (Ref.)	1 (Ref.)
Yes	5	5	1.80 (0.46–7.00)	1.98 (0.50–7.78)
**Non-Hodgkin and Burkitt lymphoma**^**d**^ **(*N =*** **98)**				
**Highest parental education in household**				
Long	34	NA	1 (Ref.)	1 (Ref.)
Medium	20	NA	0.32 (0.13–0.81)	0.34 (0.13–0.85)
Short	11	NA	3.82 (0.44–33.29)	3.95 (0.45–34.50)
**Household cohabitation status**				
Parents as couple	53	26	1 (Ref.)	1 (Ref.)
Single parent	12	7	0.85 (0.29–2.45)	0.86 (0.30–2.48)
**Depression in household parents**				
No	57	27	1 (Ref.)	1 (Ref.)
Yes	8	6	0.66 (0.20–2.12)	0.64 (0.20–2.08)
**Ewing’s sarcoma**^**e**^**(*N*** **= 86)**				
**Highest parental education in household**				
Long	NA	30	1 (Ref.)	1 (Ref.)
Medium	NA	23	1.86 (0.68–5.08)	1.91 (0.70–5.24)
Short	NA	9	0.27 (0.03–2.48)	0.28 (0.03–2.57)
**Household cohabitation status**				
Parents as couple	20	50	1 (Ref.)	1 (Ref.)
Single parent	4	12	0.76 (0.21–2.71)	0.75 (0.21–2.68)
**Depression in household parents**				
No	20	53	1 (Ref.)	1 (Ref.)
Yes	4	9	1.15 (0.31–4.28)	1.26 (0.34–4.75)
**Osteosarcoma**^**e**^ **(*N =*** **62)**				
**Highest parental education in household**				
Long	NA	19	1 (Ref.)	1 (Ref.)
Medium	NA	22	1.66 (0.43–6.49)	1.68 (0.43–6.57)
Short	NA	6	2.25 (0.37–13.58)	2.36 (0.39–14.32)
**Household cohabitation status**				
Parents as couple	11	39	1 (Ref.)	1 (Ref.)
Single parent	4	8	1.86 (0.46–7.53)	1.86 (0.46–7.58)
**Depression in household parents**				
No	NA	38	1 (Ref.)	1 (Ref.)
Yes	NA	9	0.72 (0.13–3.82)	0.70 (0.13–3.76)
**Rhabdomyosarcoma**^**e**^ **(*N*** **= 40)**				
**Highest parental education in household**				
Long	NA	NA	1 (Ref.)	1 (Ref.)
Medium	NA	NA	0.33 (0.03–3.23)	0.35 (0.04–3.49)
Short	NA	NA	2.04 (0.24–17.03)	2.16 (0.25–18.41)

^a^Model 1 is adjusted for sex, diagnosis period and diagnosis age (as a continuous variable), highest parental education in the household and household cohabitation status.

^b^Model 2 is adjusted for sex, diagnosis period and diagnosis age (as a continuous variable), highest parental education in the household, household cohabitation status, and additionally adjusted for number of total contacts in the last 24 months before diagnosis (as a continuous variable).

^c^Advanced stage: WBC at diagnosis ≥100 × 10^9^/L; Low stage: WBC at diagnosis < 100 × 10^9^/L

^d^Advanced stage: Stage 3–4; Low stage: Stage 1–2; ^e^Advanced stage: Metastases; Low stage: No distant metastases/unknown. For rhabdomyosarcoma, few observations in analyses of cohabitation status and depression, resulted in too uncertain estimates and are not shown

## Discussion

In this unique nationwide register-based study with minimal risk of bias, there seems to be an association between several indicators of family SEP, parental health and prediagnostic health care contacts among children diagnosed with cancer in Denmark. Children from households with short parental education, mixed affiliation to the work market or a parent with depression had a higher likelihood of frequent contacts overall in the last 3 months before diagnosis when compared to children with parents who have long education, are working and are not treated for depression. Further, our analyses indicate increased use of emergency health care among families with short education, mixed affiliation to work market and non-Western origin in the last 3 months before diagnosis and point towards possible differences in utilization and understanding of the health care system by socioeconomic group in Denmark. Reassuringly, we found no convincing evidence for an association between SEP and stage at diagnosis and our findings do not indicate that children of lower SEP are of higher risk of more advanced disease at diagnosis.

Socioeconomic differences in the number of prediagnostic health care contacts were also found in an earlier Danish population-based study, which showed that children with cancer had more contacts than their cancer-free peers and further that children with cancer from low- and medium-income families had higher odds of frequent contacts to their GP compared to high-income families the last 3 months prior to diagnosis [19]. Taking together, these observations and our findings point towards possible differences in understanding of the health care system by SEP. The knowledge and skills attained through e.g. education may help parents in decisions about care seeking, and to communicate, negotiate and access appropriate health services [37]. An increased contact frequency among families with low SEP may thus be due to socioeconomic differences in health literacy and/or a social gradient in the communication between families and health care professionals [44, 45]. Indeed research has shown that SEP of the parents may influence the clinicians’ approach; how they involve the parents in the clinical setting [16], and which themes are addressed in the consultation [45]. The doctor-patient/parent relationship in the prediagnostic period of childhood cancer has been explored in qualitative studies [13, 16, 45–47] including parents with different level of SEP describing differences in proactive and strategic roles in the process of obtaining a diagnosis [13, 46, 47]. A recent Danish interview study (*N* = 46) described this as a mechanism where some parents take advantage of their health-relevant cultural capital [48] in the process of obtaining a diagnosis. The strategic approach enabled them to negotiate with their GP, e.g. about further diagnostic tests and thereby speeding up the process of obtaining a diagnosis [47]. As health-relevant cultural capital is related to the SEP [48] of individuals, this mechanism may contribute to explaining our findings that children with parents of short education, looser affiliation to the work market and with depression have a higher risk of frequent contacts prior to diagnosis.

We observed no differential contact pattern by SEP in the (probably) pre-symptomatic period where number of contacts were similar and stable across SEP groups up to about 3 months prior to diagnosis at cancer group level. A pattern of more frequent contacts and likely less optimal diagnostic trajectories among groups of low SEP have been seen in general outside the cancer setting among children of parents with short education [49], children of immigrant parents [49, 50] and children with parental depression [51], while our results indicate that disparities may be confined to the presumed symptomatic period leading to diagnosis. Despite an association between childhood cancer and alert symptoms presented in general practice [52], a registry study from the UK [52], as well as a questionnaire study among Danish GPs (*n* = 363) [53], found alert symptoms to be relatively uncommon among these children. With few cases yearly, neither the GP, the GP on call nor health care professionals at the emergency departments may encounter a case of childhood cancer throughout their career. Likewise, parents most commonly have no experience with the symptoms of childhood cancer.

A significant strength of our study is the use of reliable population-based register data with almost complete coverage, not influenced by self-reports or non-participation which enables epidemiological research of high validity [54]. The complete registration independent of study hypothesis enabled us to include virtually all children diagnosed with cancer in 1998–2016 in Denmark with only few exclusions and thus minimal risk for selection bias. Annual information on SEP indicators minimized the risk of information bias often noted in self-reported data and provided us with comprehensive socioeconomic information. The inclusion of prediagnostic contacts of both primary and secondary care in our study and an explicit focus on the acute setting supports and extends the knowledge about health care use and socioeconomic disparities before diagnosis of childhood cancer.

Another strength of our study concerns the possibility to measure SEP on a household basis and not the one-sided view of the socioeconomic resources of either parent. We did not differentiate between whether the child was living with the father or the mother if living with a single parent. As according to Danish law, inhabitants, including children, can only be registered at one address although separated parents commonly share child custody in Denmark and children may live at two households, we were only able to capture the household information the child was registered at and we applied the socioeconomic and health information of the household parents based on the assumption that this represents the family circumstances of the child. Anyhow, in Denmark, all health care services for children are free in both the primary and secondary health care system and the parent’s social or health resources should formally not be a barrier for bringing the child to health care services.

Further, we chose to not mutually adjust analyses for all SEP and health variables included in the study, acknowledging that the influence of each factor may be partly overlapping, but also may represent different aspects of socioeconomic resources’ influence on health care use. To our knowledge, this is the first assessment of SEP in relation to prediagnostic health care contacts and stage of disease at diagnosis in children with cancer. Using data from the DCCR [32] enabled the application of the Toronto Guidelines to define reasonable and consistent measures of stage in children with certain types of cancer suitable for epidemiological analysis [43, 55] although groups are small even in this nationwide study. The guidelines were applicable for most cancer types except for ALL, where we used WBC as a proxy of stage [30, 56]. As the available clinical data was not always complete, accessing patient files would have provided us with more complete and accurate data to assign stage [43]. It would also have enabled us to assign stage of CNS tumors since particularly for CNS tumors sufficient information was not available from the DCCR. On the other hand, the DCCR is a comprehensive register evaluated yearly and the completeness is estimated to be 100% for children < 15 years of age at diagnosis since 2003 [32]. We have no reason to believe that missing information on stage was associated with SEP. As childhood cancer is a group of heterogeneous diseases, numbers of children in each subgroup are low reducing power even in a nationwide cohort, and at the same time we carried out a number of analyses thus chance findings cannot be ruled out. We therefore apply emphasis on patterns of estimates rather than focusing solely on the statistically significant findings.

Our findings indicate that family circumstances like parental education, affiliation to the work market, and depression are associated with increased overall utilization of the health care system before diagnosis of childhood cancer while additionally to these SEP indicators also being of non-Western origin was associated with increased utilization of acute health care. Reassuringly, we found no evident association between indicators of SEP and stage of disease among the investigated cancer types. Further research is needed in order to identify how targeted interventions can be implemented to support certain families who struggle to navigate through the health care system and are at a higher risk of experiencing less optimal prediagnostic trajectories. In future studies, more attention should be given to understanding the specific challenges these groups may experience when navigating through the health care system and their specific needs should be addressed. Finally, the long-term impact of those interventions on survival from childhood cancer should be evaluated.

Achieving timely diagnosis is dependent on early referral to secondary care. There may be a potential for reducing the number of contacts and the time to diagnosis by ensuring support for families who struggle to navigate through the health care system when their child is sick. Clinicians should pay attention to an increase in contact frequency as frequent contacts may be associated with childhood cancer [52] or other severe diseases [57]. Awareness campaigns such as Head Smart targeted parents and clinicians have proven successful in reducing time to diagnosis of brain cancer [58] and may also be considered for other cancer types. In general, communication material with instructions about diagnostic procedures, disease and treatment should however, be developed with awareness of differences in health literacy and cultural background in mind.

## Supplementary Information

**Additional file 1: Supplementary Table 1** Number of all contacts and emergency contacts to the health care system in children diagnosed with cancer 0–24 months before diagnosis by time before diagnosis and diagnostic group.

## Data Availability

The data that support the findings of this study were accessed remotely on a secure platform at Statistics Denmark. Any access to data requires permission from Statistics Denmark and the Danish Cancer Society: Danish Cancer Society Research Center, Strandboulevarden 49, 2100 Copenhagen, Denmark. Contact Person: Professor Susanne Oksbjerg Dalton. Email: sanne@cancer.dk.
