# Supplementary material for: Socioeconomic position and prediagnostic health care contacts in children with cancer in Denmark: a nationwide register study
Source: BMC Cancer. 2021 Oct 14;21:1104. doi: 10.1186/s12885-021-08837-x (PMC8518314; doi:10.1186/s12885-021-08837-x)
Supplement: Supplementary file 1 — Additional file 1: Supplementary Table 1 Number of all contacts and emergency contacts to the health care system in children diagnosed with cancer 0–24 months before diagnosis by time before diagnosis and diagnostic group. [file 12885_2021_8837_MOESM1_ESM.docx]

| **Supplementary table 1: Number of all contacts and emergency contacts to the health care system in children diagnosed with cancer 0-24 months before diagnosis by time before diagnosis and diagnostic group.** | | | | | | | | | | |
| --- | --- | --- | --- | --- | --- | --- | --- | --- | --- | --- |
|  | **Leukemia** | | **Lymphoma** | | **CNS tumors** | | **Bone tumors and soft tissue sarcomas** | | **Other non-CNS solid tumors** | |
|  | **(N=905)** | | **(N=314)** | | **(N=805)** | | **(N=321)** | | **(N=698)** | |
| **Contacts** | **Median** | **Range** | **Median** | **Range** | **Median** | **Range** | **Median** | **Range** | **Median** | **Range** |
| **All contacts (mts)** |  |  |  |  |  |  |  |  |  |  |
| 0-3 mts | 9 | 0-54 | 8 | 0-31 | 6 | 0-33 | 7 | 0-35 | 6 | 0-40 |
| 4-6 mts | 1 | 0-24 | 1 | 0-37 | 2 | 0-23 | 1 | 0-17 | 2 | 0-22 |
| 7-12 mts | 3 | 0-77 | 2 | 0-36 | 3 | 0-47 | 2 | 0-43 | 4 | 0-35 |
| 13-18 mts | 3 | 0-49 | 2 | 0-36 | 3 | 0-40 | 2 | 0-31 | 3 | 0-35 |
| 19-24 mts | 3 | 0-50 | 2 | 0-36 | 3 | 0-50 | 2 | 0-31 | 3 | 0-47 |
| 0-24 mts | 23 | 0-182 | 19 | 0-116 | 19 | 0-151 | 16 | 0-134 | 18 | 0-114 |
| **Emergency contacts (mts)** |  |  |  |  |  |  |  |  |  |  |
| 0-3 mts | 2 | 0-14 | 1 | 0-17 | 1 | 0-15 | 1 | 0-10 | 1 | 0-19 |
| 4-6 mts | 0 | 0-18 | 0 | 0-7 | 0 | 0-10 | 0 | 0-5 | 0 | 0-11 |
| 7-12 mts | 0 | 0-27 | 0 | 0-15 | 0 | 0-17 | 0 | 0-7 | 0 | 0-15 |
| 13-18 mts | 0 | 0-18 | 0 | 0-17 | 0 | 0-15 | 0 | 0-10 | 0 | 0-16 |
| 19-24 mts | 0 | 0-17 | 0 | 0-11 | 0 | 0-22 | 0 | 0-12 | 0 | 0-9 |
| 0-24 mts | 3 | 0-49 | 2 | 0-40 | 2 | 0-56 | 2 | 0-29 | 2 | 0-37 |
| Mts: Months before diagnosis; Range: Minimum and maximum number of contacts in the specific time interval. | | | | | | | | |  |  |
